# Enhancement of thermoalkaliphilic xylanase production by *Pichia pastoris* through novel fed-batch strategy in high cell-density fermentation

**DOI:** 10.1186/s12896-017-0361-6

**Published:** 2017-06-21

**Authors:** Tingting Shang, Dayong Si, Dongyan Zhang, Xuhui Liu, Longmei Zhao, Cong Hu, Yu Fu, Rijun Zhang

**Affiliations:** 1 Laboratory of Feed Biotechnology, State Key Laboratory of Animal Nutrition, College of Animal Science & Technology, China Agricultural University, No.2 Yuanmingyuan West Road, Haidian District Beijing, 100193 China; 2 College of Animal Science and Technology, Henan University of Science and Technology, Luoyang, 471003 China

**Keywords:** Thermoalkaliphilic xylanase, *Pichia pastoris*, Fermentation parameter optimization, Bioprocess, Fed-batch strategy, High cell-density fermentation

## Abstract

**Background:**

Xylanase degrades xylan into monomers of various sizes by catalyzing the endohydrolysis of the 1,4-β-D-xylosidic linkage randomly, possessing potential in wide industrial applications. Most of xylanases are susceptible to be inactive when suffering high temperature and high alkaline process. Therefore, it is necessary to develop a high amount of effective thermoalkaliphilic xylanases. This study aims to enhance thermoalkaliphilic xylanase production in *Pichia pastoris* through fermentation parameters optimization and novel efficient fed-batch strategy in high cell-density fermentation.

**Results:**

Recombinant xylanase activity increased 12.2%, 7.4%, 12.0% and 9.9% by supplementing the *Pichia pastoris* culture with 20 g/L wheat bran, 5 mg/L L-histidine, 10 mg/L L-tryptophan and 10 mg/L L-methionine in shake flasks, respectively. Investigation of nutritional fermentation parameters, non-nutritional fermentation parameters and feeding strategies in 1 L bioreactor and 1 L shake flask revealed that glycerol and methanol feeding strategies were the critical factors for high cell density and xylanase activity. In 50 L bioreactor, a novel glycerol feeding strategy and a four-stage methanol feeding strategy with a stepwise increase in feeding rate were developed to enhance recombinant xylanase production. In the initial 72 h of methanol induction, the linear dependence of xylanase activity on methanol intake was observed (R^2^ = 0.9726). The maximum xylanase activity was predicted to be 591.2 U/mL, while the actual maximum xylanase activity was 560.7 U/mL, which was 7.05 times of that in shake flask. Recombinant xylanase retained 82.5% of its initial activity after pre-incubation at 80 °C for 50 min (pH 8.0), and it exhibited excellent stability in the broad temperature (60–80 °C) and pH (pH 8.0–11.0) ranges.

**Conclusions:**

Efficient glycerol and methanol fed-batch strategies resulting in desired cell density and xylanase activity should be applied in other *P. pastoris* fermentation for other recombinant proteins production. Recombinant xylanases with high pH- and thermal-stability showed potential in various industrial applications.

## Background

Xylan is the major component of hemicellulose, and it is the second most abundant renewable resource [1]. Xylanase (β-1,4-Endoxylanases, EC 3.2.1.8) degrades xylan into monomers by catalyzing the endohydrolysis of the 1,4-β-D-xylosidic linkage between molecules of xylose in the main chain randomly [2]. Lignocellulosic matter consists of ca. 40% cellulose, 33% hemicellulose, and 23% lignin by dry weight [3]. Endoxylanases play an important role in bioconversion of lignocellulose in feed, food, biofuel, pulp and paper industry and are widely used as raw materials in lots of industrial processes [2, 4].

Thermoalkaliphilic xylanases are required in detergent, textile, paper and pulp industries. With them, enzymes hydrolysis will be performed at elevated temperatures without cooling substrate down, thus process time will be shortened, energy will be saved and saccharification yield can be improved, leading to a more economical and feasible process [5]. Up to now, a large number of xylanases have been produced and investigated, however, most of xylanases are susceptible to denaturation when exposed to the high temperature and high alkaline process [6]. Therefore, the development of a high amount of effective thermoalkaliphilic xylanases is highly desirable.

With the development of recombinant protein engineering, genetic engineering strains are employed to produce recombinant xylanase effectively. The methylotrophic yeast *Pichia pastoris* (*P. pastoris*), one of the most extensively used expression systems for heterologous proteins production, has many advantages: (1) generally strong and tightly regulated promoters, (2) available molecular manipulation tools, (3) high cell-density fermentation on inexpensive substrate, (4) protein production in secretory fashion [7, 8]. Unlike extremozyme production by extremophile under unusual condition, the bioprocess of extremozyme production by mesophilic host organisms such as *P. pastori*s can be optimized for high biomass and enzyme activity. Therefore, expression of thermoalkaliphilic xylanases in *P. pastori*s is attractive.

Different micro-organisms have different physiological phenotype and biosynthetic capacity, thus fermentation parameters including growth medium composition and cultivation conditions (pH, temperature, dissolved oxygen (DO), etc.) affect producing cells growth and recombinant xylanase production [9]. The levels of recombinant protein production in *P. pastoris* in bioreactor cultures are typically much higher than those in shake-flask cultures [10], since these factors including pH, temperature, oxygen transfer and substrate addition can be monitored and controlled simultaneously. In bioreactor cultures, *P. pastoris* can grow to high cell density (exceeding 100 g/L dry cell weight (DCW)) [11, 12], and a large amount of recombinant proteins production is based on high concentration of producing cells in the culture [13]. For *P. pastoris* expression system, alcohol oxidase 1 (AOX1) promoter has been the most widely reported and utilized, which can be regulated by methanol for recombinant proteins production [14]. Therefore, cultivation of *P. pastoris* is normally separated into three phases: batch phase, where cells grow until initial carbon source has been exhausted; fed-batch phase, where the same carbon source is fed for high cell density; induction phase, recombinant protein is induced to produce by feeding inducer after initial carbon source exhaustion [15–17]. Nutrient feeding strategy is critical for recombinant proteins production by *P. pastoris* in high cell-density fermentation. Glycerol is a common initial carbon source, while it may repress the AOX1 promoter and lower recombinant proteins production [18, 19]. The inducer methanol excessing a certain level is cytotoxic and inhibits cells growth [20]. To prevent overfeeding of nutrients, feedback-controlled feeing systems are developed, in which the nutrient is added according to a change in DO, pH, substrate concentration or by-product (often has negative effects on targeted recombinant protein production) concentration [21–24].

In previous study, the *xynA* xylanase gene from *Thermobiafida fusca* YX was cloned and expressed in *P. pastoris* X-33. *Thermobifida fusca* is a thermophilic actinomycete and a major degrader of plant cell walls in heated organic materials. This recombinant xylanase was optimally active at 80 °C and pH 8.0 [25]. In the present study, we optimized fermentation parameters and developed novel glycerol and methanol fed-batch strategies to enhance recombinant thermoalkaliphilic xylanase production in *P. pastoris* in high cell-density fermentation in 50 L bioreactor.

## Methods

### Strains and reagents

To obtain the secreted thermoalkaliphilic xylanase, a *P. pastoris* strain X-33 transformed with pPICZɑ-A vector bearing the construct *xynA* of *Thermobifida fusca* YX with a C-terminal histidine tag locates at the downstream of AOX1 promoter was used, and the xylanase was optimally active at 80 °C and pH 8.0 [25]. Yeast extract and peptone were obtained from OXOID (Basingstoke, Hampshire, UK), yeast nitrogen base (YNB) without amino acids was purchased from BD (Sparks, MD, USA), biotin and phenylmethylsulfonyl fluoride (PMSF) were obtained from Amresco (Solon, OH, USA). Xylan from beechwood was purchased from Sigma Chemical Company (St. Louis, MO, USA). Xylose was from Wako (Osaka, Japan). Corn bran, corncob, cottonseed hull and wheat bran were purchased from Xiangchi (Shandong, China), Chunjing (Guangdong, China), Hengfeng Mining (Hebei, China) and Zhongnongzhenya (Beijing, China), respectively. All of the lignocellulosic materials passed through 0.2-mm screen sieve. Other chemicals were obtained from Sinopharm Chemical Reagent Co., Ltd (Shanghai, China).

### Cultivation of recombinant *P. pastoris* in shake flask

Yeast extract peptone dextrose (YPD) plates for seed cultivation contained 10 g/L yeast extract, 20 g/L peptone, 20 g/L dextrose and 20 g/L agar. A single colony was inoculated in 50 mL buffered glycerol-complex medium (BMGY, 10 g/L yeast extract, 20 g/L tryptone, 13.4 g/L YNB, 4 × 10^−4^ g/L biotin, 100 mM potassium phosphate (pH 6.0), 1% (v/v) glycerol), and incubated at 30 °C, 220 rpm for 24 h in a 500 mL baffled shake flask, then 5 mL of the culture was transferred into 100 mL BMGY medium in 1 L baffled shake flask and cultured at the same condition. To select the optimal carbon source, xylanase activity and DCW were determined with 1% (w/v) glucose or 1% (w/v) maltose as carbon source to replace 1% (v/v) glycerol in the BMGY medium. After incubation for 60 h, 0.5% (v/v) methanol was added every 24 h. The culture was sampled at 0 h and 24 h of fermentation, and then every 12 h. We also investigated the xylanase activity and DCW with 1% (v/v) glycerol or 1% (w/v) glucose as the initial carbon source, and 0.5% (v/v) methanol was added every 24 h after inoculation. To obtain highly active xylanase, 2% (w/v) tryptone in the BMGY medium was replaced by various nitrogen sources (2% (NH_4_)_2_SO_4_, 2% (NH_2_)_2_CO, 2% soya peptone, 2% (v/v) ammonia solution (pH 6.0)). To study the effect of amino acid, protease inhibitor and lignocellulosic material on xylanase production, recombinant *P. pastoris* was cultured in BMGY medium supplemented with 5 mg/L L-histidine, 10 mg/L L-tryptophan, 10 mg/L L-methionine, 1 mM PMSF, 1 mM ethylenediaminetetraacetic acid (EDTA), 20 g/L corn bran, 20 g/L corncob, 20 g/L cottonseed hull, 20 g/L wheat bran, 10 g/L corn bran + 10 g/L wheat bran, 10 g/L corncob + 10 g/L wheat bran, and 10 g/L cottonseed hull + 10 g/L wheat bran for 96 h, respectively. Methanol (0.5% (v/v)) was added every 24 h to maintain induction. Samples were collected at 96 h for xylanase activity analysis.

### Cultivation of recombinant *P. pastoris* in 1 L bioreactor

Seed culture was prepared in BMGY medium at 30 °C, 220 rpm in baffled shake flask for 24 h. Seed culture (30 mL) was inoculated to 600 mL BMGY medium supplemented with 3.0 mg L-histidine, 6.0 mg L-tryptophan, 6.0 mg L-methionine and 12.0 g wheat bran in 1 L bioreactor. Cultivation was conducted at 30 °C, 800 rpm. The pH was maintained at 6.0 with 28% (w/v) NH_4_OH and DO was kept above 20%. Different treatments were performed: I) for 1 L bioreactors, the glycerol growth phase was maintained for 24 h by feeding 1% (v/v) glycerol at 9 h and 17 h, the methanol induction phase was maintained for following 120 h by feeding 0.5% (v/v) methanol at inoculated time of 24 h, 32 h, 48 h, then every 8 h to the end; II) for the other 1 L bioreactors, 0.5% (v/v) methanol was added every 24 h after inoculation; III) *P. pastoris* expressed xylanase in 100 mL BMGY medium without supplementation of amino acids and wheat bran in 1 L shake flasks at 30 °C, 220 rpm, but having the same glycerol and methanol feeding strategies with treatment II.

### Cultivation of recombinant *P. pastoris* in 50 L bioreactor

Seed culture (1.25 L) was inoculated to 25 L basal salt medium as Li et al. described, and (NH_4_)_2_SO_4_ was replaced by (NH_2_)_2_CO in a 50 L bioreactor (Baoxing Co., Shanghai, China) [26]. In addition, we added 0.125 g L-histidine, 0.25 g L-tryptophan, 0.25 g L-methionine and 500 g wheat bran into the 50 L bioreactor. Temperature was maintained at 30 °C by water cooling, pH was automatically adjusted to 6.0 with 28% (w/v) NH_4_OH, the agitation rate ranged from 200 to 350 rpm, and the tank pressure was 0.7 bar. A total cultivation time of 120 h included glycerol growth stage (0–24 h) and methanol induction stage (25–120 h). Glycerol cultivation was divided into three phases: batch phase (0–19 h): cells grew until the initial carbon source glycerol was depleted, which was indicated by a sudden increase in DO; fed-batch phase (20–22 h): 50% (v/v) glycerol containing 1.2% (v/v) PTM1 trace salt solution was fed at the rate of 10.4 mL h^−1^ L^−1^; starvation phase (23–24 h): glycerol feeding was stopped, and depletion of glycerol was indicated by DO rose to 100%. For methanol induction, feeding manner of 100% (v/v) methanol containing 1.2% (v/v) PTM1 trace salt solution was shown in the Table 1. Samples were collected every 12 h.

**Table 1:** Methanol feeding rate in methanol induction phase

	Methanol feeding rate (mL h^−1^ L^−1^)			
	0.8	1.2	1.6	1.8
Induction time (h)	0–12	13–24	25–48	49–96

### Determination of DCW

Five milliliters of culture broth was placed in pre-weighed tube and centrifuged at 12,000 rpm for 5 min. The supernatant was removed and stored at −20 °C for xylanase activity analysis. The tube was dried at 100 °C to a constant weight, and then DCW was calculated and reported as g/L (g DCW per L of culture).

### Determination of xylanase activity

The xylanase activity was determined by measuring the reducing sugar released from beechwood xylan with dinitrosalicylic acid (DNS) method [27, 28]. The reaction mixture contained 20 μL of appropriately diluted crude enzyme solution and 180 μL of 1% (w/v) beechwood xylan in 50 mM phosphate buffer (pH 8.0). After being incubated at 80 °C for 20 min, the reaction was terminated by chilling the mixture on ice and adding 200 μL of DNS solution. After boiling sample for 10 min, the absorbance of 200 μL sample at 540 nm was measured by iMark Microplate Reader (Bio-Rad, Hercules, CA, USA). One unit of xylanase activity was defined as the amount of xylanase releasing 1 μmole of reducing sugar per minute under the assay condition (with xylose as a standard).

### Characterization of the recombinant xylanase

To determine the effect of pH on xylanase stability, xylanase was pre-incubated at 50 °C in different pH buffers (pH 8.0 using sodium phosphate buffer, pH 9.0–11.0 using glycine-NaOH buffer) for 0, 1, 2, 3, 5, 7, 9, 11 h. Samples were taken at different time intervals and xylanase residual activity was measured according to the above method after cooling.

To evaluate thermal stability, xylanase was pre-incubated at 60 °C, 70 °C, 80 °C and 90 °C for 0, 5, 10, 15, 20, 25, 30, 40, 50 and 60 min. Samples were taken at different time intervals. After cooling, residual activity of treated xylanase was assayed according to the above method.

### Statistical analysis

The SPSS 17.0 statistical software package and software Microsoft Excel 2010 were used to analyze and plot the experimental data. The data were expressed as the means ± standard deviations. The differences among the groups were analyzed by one-way analysis variance (ANOVA) followed by Duncan’s method. A *p*-value less than 0.05 was considered statistically significant.

## Results and discussion

### Effect of carbon source on DCW and xylanase activity in shake flask

**Fig. 1:**
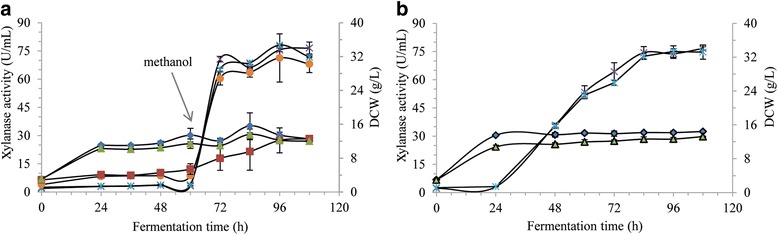
DCW and xylanase activity profiles with xylanase expression at different carbon sources in shake flask. **a** methanol was added after fermentation for 60 h, **b** methanol was added every 24 h. Circles, xylanase activity (maltose); asterisks, xylanase activity (glucose); crosses, xylanase activity (glycerol); triangles, DCW (glucose); squares, DCW (maltose); diamonds, DCW (glycerol)

Carbon source provides energy and carbon skeletons for *P. pastoris* cells growth and recombinant protein production. The xylanase activity was almost zero before induction with glycerol or glucose as initial carbon source (Fig. 1a). It meant that *P. pastoris* cells growth could be supported by glycerol or glucose, however, xylanase gene expression driven by AOX1 promoter can’t be induced in glycerol- or glucose-containing medium, which was in agreement with Hellwig’s report [29]. When 0.5% (v/v) methanol was added at 60 h, the xylanase activity increased in the medium whose initial carbon source was glucose or glycerol, and it was always higher than that in the medium with maltose as initial carbon source. There was almost no change in DCW after fermentation for 24 h in glycerol- or glucose-grown *P. pastoris* cultivation, while the DCW was lower at the beginning of fermentation and increased with the time in maltose-grown *P. pastoris* cultivation (Fig. 1a). We also investigated the DCW and xylanase activity in glycerol- and glucose-grown *P. pastoris* cultivation when 0.5% (v/v) methanol was added every 24 h after inoculation (Fig. 1b), and the maximum xylanase activity in both glycerol-grown and glucose-grown *P. pastoris* cultivation detected at 84 h was about 75.0 U/mL and kept more or less the same until 108 h. After fermentation for 24 h, the DCW ranged from 13.53 g/L to 14.43 g/L with glycerol as initial carbon source, and it ranged from 10.75 g/L to 13.23 g/L with glucose as initial carbon source (Fig. 1b). Glucose, one of fermentative carbon sources, tends to be avoid in favor of the non-fermentative carbon source – glycerol, because the by-product ethanol may repress the AOX1 promoter for recombinant protein production [10, 30]. In this study, glycerol was treated as the optimal initial carbon source.

### Supplementation of wheat bran improved xylanase activity in shake flask

**Fig. 2:**
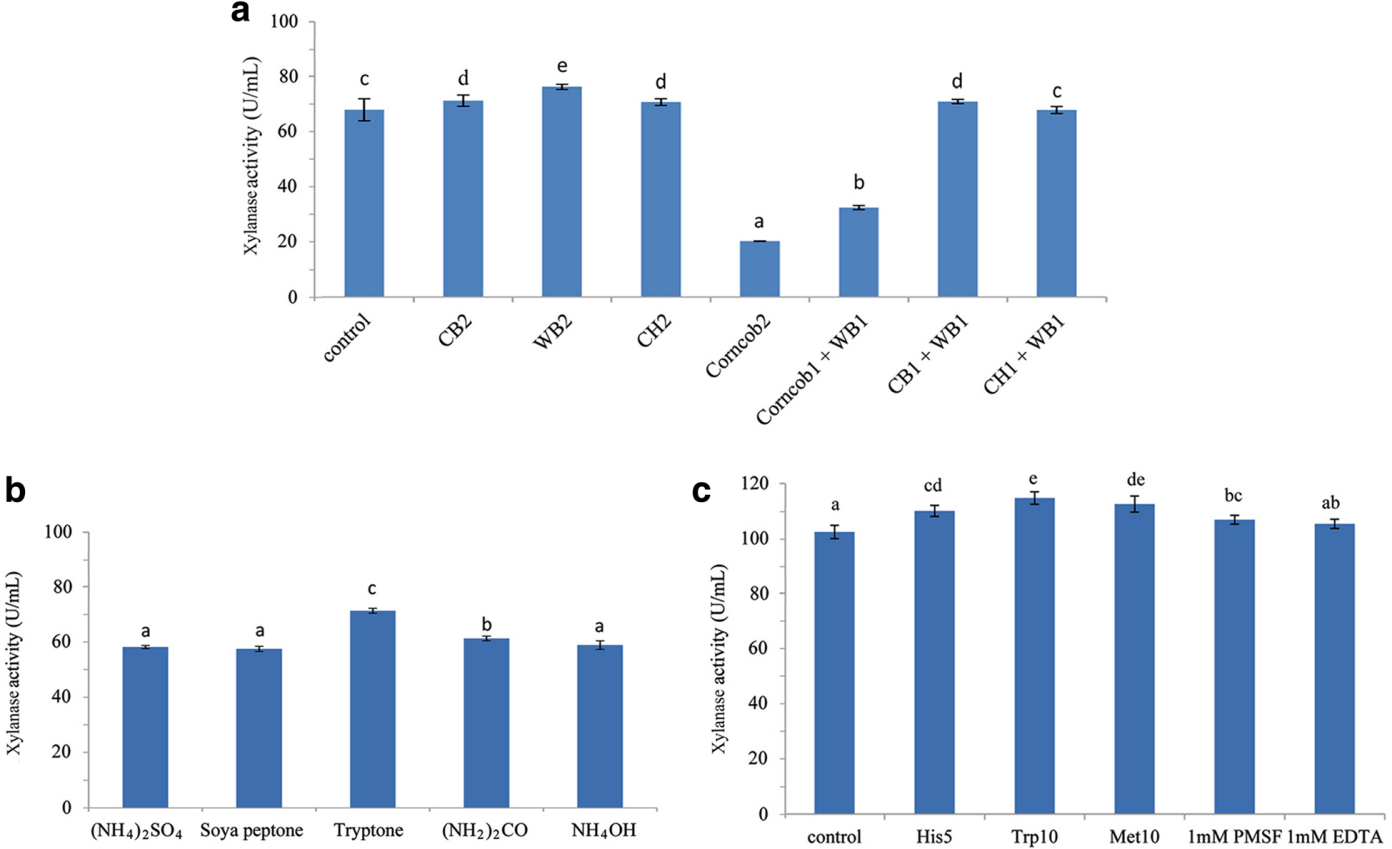
Effect of fermentation parameters on xylanase activity in shake flask. **a** lignocellulosic materials, **b** nitrogen sources, **c** amino acids and protease inhibitors. CB2, corn bran (20 g/L); WB2, wheat bran (20 g/L); CH2, cottonseed hull (20 g/L); Corncob2, corncob (20 g/L); Corncob1 + WB1, corncob (10 g/L) and wheat bran (10 g/L); CB1 + WB1, corn bran (10 g/L) and wheat bran (10 g/L); CH1 + WB1, cottonseed hull (10 g/L) and wheat bran (10 g/L); His5, L-histidine (5 mg/L); Trp10, L-tryptophan (10 mg/L); Met10, L-methionine (10 mg/L)

Lignocellulosic material contains a plenty of xylan and other nutrients. It may serve as substrate for cells growth or inducer for hemicellulose enzyme production, [31]. BMGY medium supplemented with lignocellulosic materials (corn bran, wheat bran, cottonseed hull and corncob) was used for xylanase production in *P. pastoris*. The xylanase activity was significantly improved by supplementing 2% (w/v) wheat bran (Fig. 2a). Wheat bran can provide high quality proteins, minerals, and phenolic and bioacitve carbohydrate after enzymatical hydrolysis [32–34], which may be benefit for *P. pastoris* cells growth and recombinant xylanase production. In previous studies, corncob could be used by filamentous fungi (*Rhizopus stolonifer* JS-1008, *Streptomyces sp.* CS802, *Aspergillus niger* CECT 2700, etc.) for xylanase production [35–37]. However, the xylanase activity was decreased by 70.31% with adding 2% (w/v) corncob to *P. pastoris* culture in this study (Fig. 2a).

### Effect of nitrogen source on xylanase activity in shake flask

Nitrogen source plays an important role in the growth of micro-organisms and metabolite production. To study the effect of nitrogen source on xylanase activity, tryptone in BMGY medium was replaced by one organic nitrogen source – soya peptone and three inorganic nitrogen sources – (NH_2_)_2_CO, (NH_4_)_2_SO_4_ and NH_4_OH (pH 6.0), respectively. As shown in Fig. 2b, when tryptone was used as nitrogen source, the highest xylanase activity reached 71.3 U/mL at 96 h, and the second highest xylanase activity was 61.3 U/mL with (NH_2_)_2_CO as nitrogen source. No obvious difference in xylanase activity was observed when NH_4_OH (pH 6.0), (NH_4_)_2_SO_4_ or soya peptone was used. For economical and commercial purpose, (NH_2_)_2_CO has potential application in large-scale fermentation.

### Supplementation of amino acid and protein inhibitor improved xylanase activity in shake flask

When adding PMSF to the medium, xylanase activity was increased from 102.3 U/mL to 106.7 U/mL (*P* < 0.05), it can be inferred that extracellular proteases were produced during xylanase production in *P. pastoris*. Some researchers indicated that supplementation of amino acids improved recombinant protein production [38], and addition of casamino acids could protect protein expressed by *P. pastoris* from proteolytic degradation [39]. The xylanase activity was increased by 7.4%, 12.0% and 9.9% when supplemented BMGY medium with 5 mg/L L-histidine, 10 mg/L L-tryptophan and 10 mg/L L-methionine, respectively (Fig. 2c). Besides acting as alternative and competing substrates to decrease hydrolysis of target protein, sufficiently available exogenous amino acids in nitrogen-metabolism can be incorporared directly into biomass [40].

### Xylanase production in *P. pastoris* in 1 L bioreactor and 1 L shake flask

**Fig. 3:**
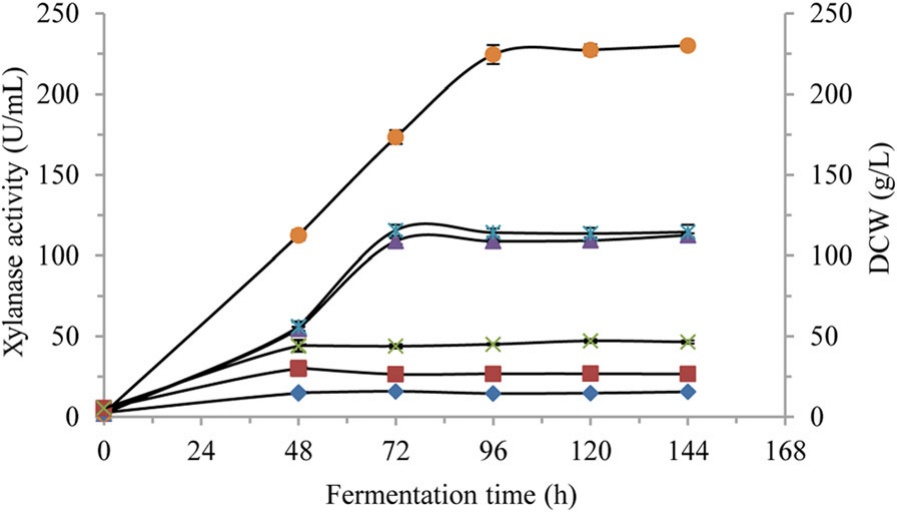
Recombinant xylanase production in *P.pastoris* in 1 L bioreactor and 1 L shake flask. Circles, xylanase activity in treatment I; asterisks, xylanase activity in treatment II; triangles, xylanase activity in treatment III; crosses, DCW in treatment I; squares, DCW in treatment II; diamonds, DCW in treatment III

**Fig. 4:**
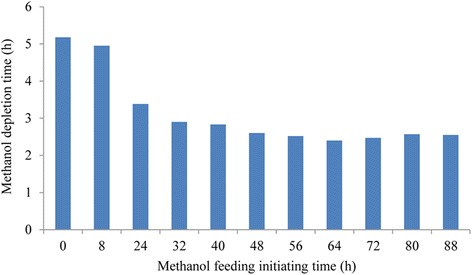
Methanol depletion time with different methanol feeding initiating time in 1 L bioreactor

Singh et al. reported that the recombinant protein produced in bioreactor was significantly higher than that in shake flask [41]. As shown in Fig. 3, DCW in all of the treatments was stable after 48 h, however, DCW in treatment I was 45.05 g/L at 96 h, significantly higher than those in treatment II (26.73 g/L) and treatment III (14.48 g/L). It can be inferred that DCW was highly correlated to glycerol content. The maximum xylanase activity reached 114.1 U/mL in treatment II and 108.8 U/mL in treatment III at 72 h, however, the maximum xylanase activity was 224.4 U/mL at 96 h in treatment I. These results obviously suggested that glycerol and methanol feeding strategies were the critical factors in high DCW and xylanase activity. In treatment I, glycerol had already been depleted at 24 h of fermentation, which was indicated by a spike in DO, and then pulsed addition of 6 ml of methanol (50% (v/v)) in the 600 mL BMGY medium was performed at 24 h, 32 h, 48 h, and then every 8 h to the end of fermentation in 1 L bioreactors. When *P. pastoris* cells were growing with sufficient carbon source, DO was strongly demanded for carbon source assimilation, as a result the DO tension fell and became very low; while the DO tension increased sharply once carbon source was depleted. According to these phenomena, the methanol depletion time from the time point pulsed addition of methanol to the time point when DO rose to 50% again was recorded and shown in Fig. 4. In general, methanol consumption rate increased in the initial 64 h and then showed a tendency of decrease, and the result also suggested methanol was insufficient in *P. pastoris* cultivation (Fig. 4).

### Development of novel glycerol and methanol fed-batch strategies in 50 L bioreactor

**Fig. 5:**
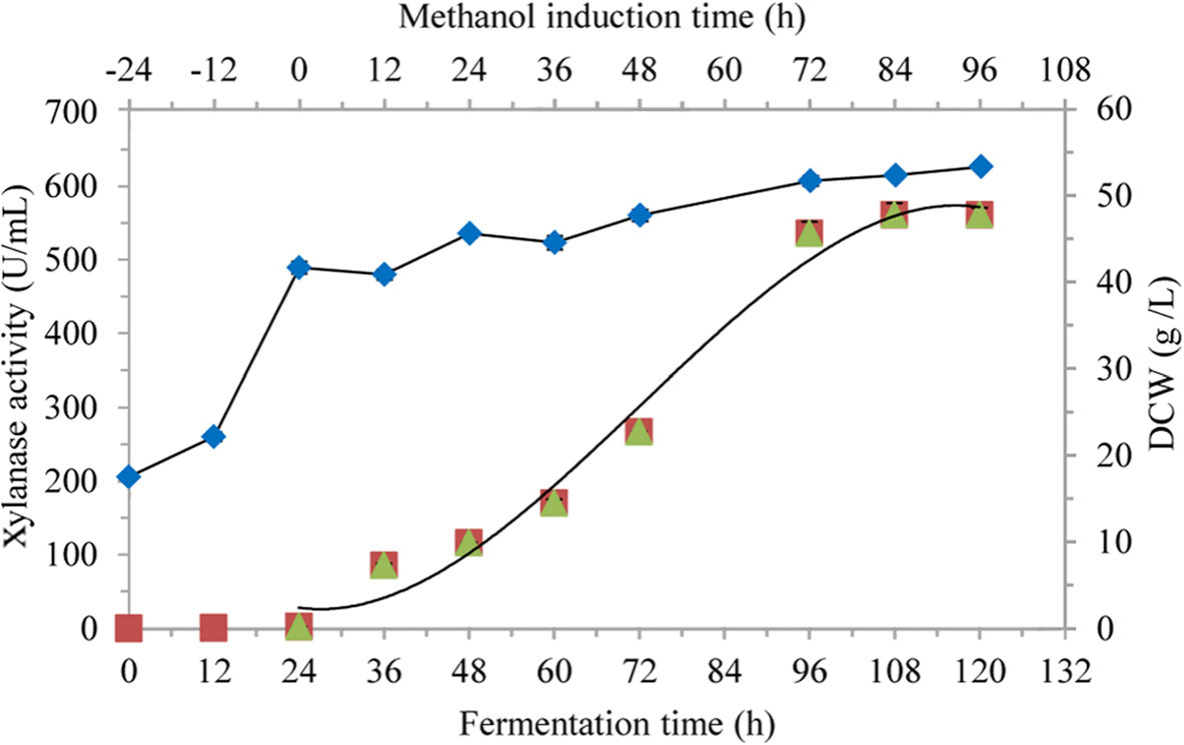
Recombinant xylanase production in *P. pastoris* in 50 L bioreactor

Based on the above findings, novel glycerol and methanol fed-batch strategies were adopted to improve DCW and xylanase activity in 50 L bioreactor, where glycerol and methanol feeding rates, DO, pH and temperature could be easily controlled and monitored. Glycerol enters glycolysis and requires respiration to oxidize NADH to serve as energy source [18]. A lower initial glycerol concentration and fed-batch operation led to a higher cell growth rate and desired biomass [23], and the concentration of the producing cells in the medium affects the volumetric productivity of recombinant enzymes [13]. In this study, glycerol cultivation (total 24 h) consisted of batch phase (0–19 h), fed-batch phase (20–22 h) and starvation phase (23–24 h). After culturing *P. pastoris* in glycerol-containing medium for 24 h, depletion of glycerol was indicated by DO rose to 100%, and the DCW reached 41.8 g/L, but almost no xylanase activity was detected (Fig. 5), which indicated AOX1 promoter could be tightly regulated by methanol, and glycerol catabolic process was relatively independent of the methanol metabolism pathways [42]. Weinhandl et al. also reported glycerol supported *P. pastoris* cells growth without inducing the AOX promoter [43], however, Bara et al. observed leaky expression from AOX promoter, and functional recombinant protein was detected in bioreactor prior to methanol induction in glycerol-grown *P. pastoris* cultivation [20]. In any case, pre-induction phase cultivation should be optimized to maximize recombinant protein production.

**Fig. 6:**
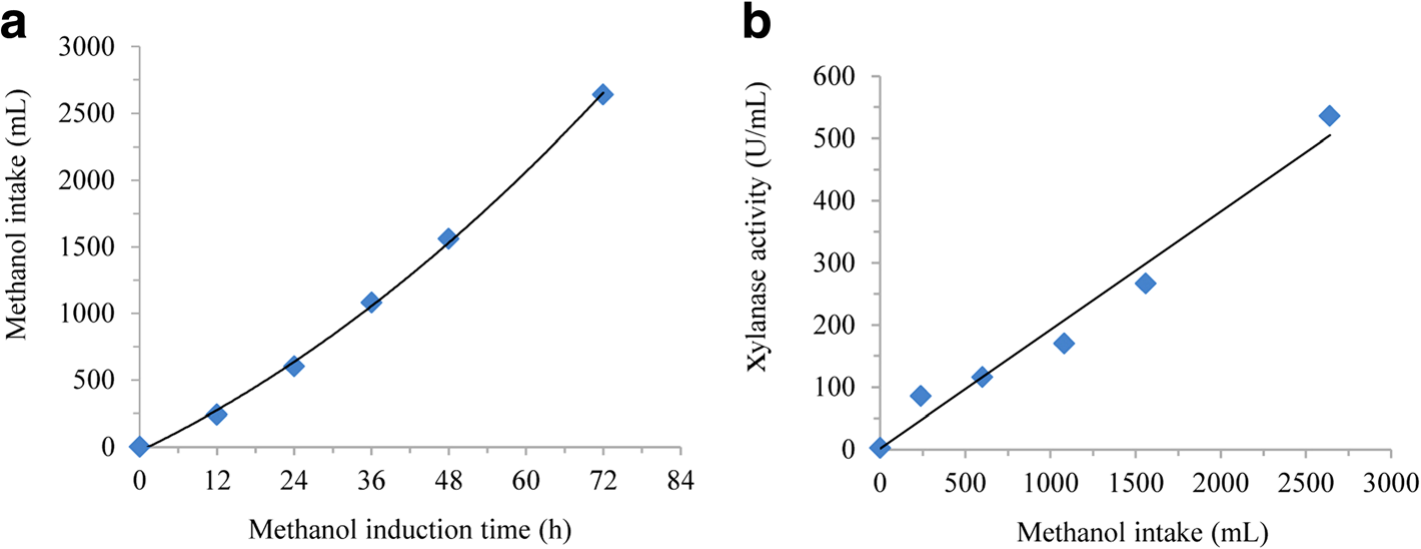
Time-course of methanol intake and dependence of xylanase activity on methanol intake in 50-L bioreactor. **a** time-course of the methanol intake, **b** linear dependence of xylanase activity on methanol intake

The methanol not only induces the AOX1 promoter in *P. pastoris* for recombinant protein production, but it is also the carbon/energy source. The methanol concentration seriously affects cell uptake rate and specific production rate. Notably, excess of methanol led to accumulation of formaldehyde to toxic level and reduced biomass yield [44]. Methanol is assimilated vigorously with DO consumption. The oxygen demand not only associates with the cellular electron transport, but alcohol oxygenase also requires molecular oxygen as a substrate [45]. In this study, methanol feeding rate was adjusted to response to online measured fermentation parameter – DO, which was kept fluctuating around fixed value. Methanol intake (feeding) rate gradually increased as described in Materials and Methods. And as shown in Fig. 6a, in the initial 72 h of methanol induction, methanol intake was adapted to the equation:

1 $$ \mathrm{M}\mathrm{I}=0.{{1984\mathrm{m}}_{\mathrm{time}}}^2+23{\mathrm{m}}_{\mathrm{time}}{\textstyle \hbox{--} }30.286\left({\mathrm{R}}^2=0.9989\right) $$

where MI was methanol intake, m_time_ was methanol induction time. The xylanase activity increased with m_time_ and finally reached a plateau (Fig. 5). It was fitted for equation:

2 $$ \mathrm{X}\mathrm{a} = -0.00{{15\ \mathrm{m}}_{\mathrm{time}}}^3 + 0.{{2184\ \mathrm{m}}_{\mathrm{time}}}^2-1.285\ {\mathrm{m}}_{\mathrm{time}} + 28.034\ \left({\mathrm{R}}^2 = 0.9842\right) $$

where Xa was xylanase activity. The predicted maximum xylanase activity was 591.2 U/mL when methanol was fed for 94.0 h according to Eq. 2 and extreme value theorem, and the actual maximum xylanase activity was 560.7 U/mL, which was 7.05 times of that in shake flask. The linear dependence of xylanase activity on methanol intake in initial 72 h of methanol induction was observed (Fig. 6b), and the resulting equation:

3 $$ \mathrm{X}\mathrm{a} = 0.1906\ \mathrm{M}\mathrm{I} + 1.5033\ \left({\mathrm{R}}^2 = 0.9726\right) $$

At the end of total cultivation, the DCW reached 53.48 g/L in 50 L bioreactor, far more than that in shake flask (Fig. 5). Both xylanase activity and DCW were significantly improved through novel glycerol and methanol fed-batch strategies in high cell-density fermentation.

### Characterization of xylanase activity under high pH and temperature

**Fig. 7:**
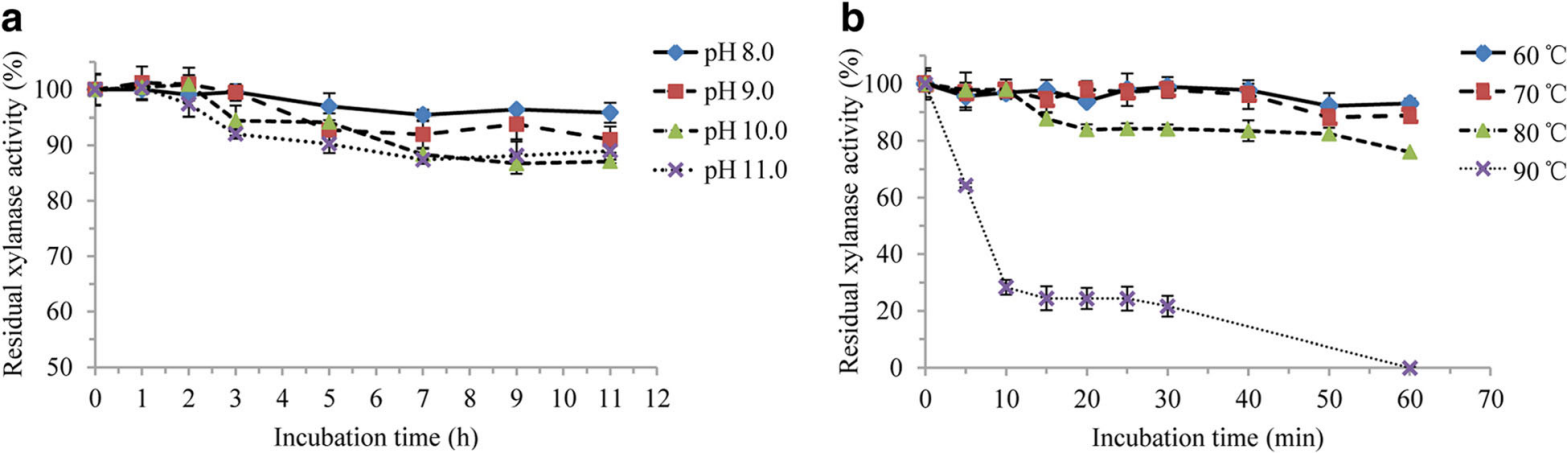
The properties of recombinant xylanase. **a** effect of pH on the stability of recombinant xylanase, **b** effect of temperature on the stability of recombinant xylanase

The optimal temperature of xylanase has been proved to be 80 °C at the optimal pH 8.0 [25]. We evaluated the residual xylanase activity at 50 °C with different incubation time in a range of pH 8.0–11.0. It showed excellent pH stability. After pre-incubating for 11 h, residual xylanase activity was over 95.0% at pH 8.0, and more than 85.0% of xylanase activity was retained even at pH 11.0 (Fig. 7a). According to Ergun and Calik, the most promising recombinant thermoalkaliphilic xylanase of *B. halodurans* TSEV1 expressed in *P. pastoris* retained 50.0% of its initial activity at 80 °C for 45 min (pH 9.0), whose xylanase activity was 502.0 U/mL [5, 46]. In this study, residual xylanase activity was still over 80.0% after pre-incubation at 80 °C for 50 min (pH 8.0); about 90.0% of its initial activity was retained at 70 °C for 60 min (pH 8.0) (Fig. 7b). This xylanase with high pH- and temperature tolerance showed potential in industry, especially in the pulp and paper industry [6]. The bioprocess will be more economical and feasible when the labor intensive pH and temperature readjustment step is trimmed down [47].

### Long-term storage stability

Enzyme stability is a critical factor for industrial application [48]. The fermentation broth was stored at −20 °C for 1 year to analyze xylanase activity. The residual xylanase activity was 99.6% after 1 year, providing important support for xylanase to apply widely.

## Conclusions

Recombinant thermoalkaliphilic xylanase production in *P. pastoris* was enhanced by fermentation parameters optimization. Glycerol and methanol feeding strategies were the critical factors leading to the difference of DCW and xylanase activity between in bioreactor and in shake flask. Novel efficient glycerol and methanol fed-batch strategies were developed to improve xylanase activity to be 560.7 U/mL in high cell-density fermentation in 50 L bioreactor, and should be applied in other *P. pastoris* fermentation for recombinant proteins production. Recombinant xylanase with high stability in the broad temperature (60–80 °C) and pH (pH 8.0–11.0) ranges was suitable for various industrial applications such as detergent, textile, and paper and pulp industry.

## Abbreviations

AOX1: Alcohol oxidase 1
BMGY: Buffered glycerol-complex medium
DCW: Dry cell weight
DNS: Dinitrosalicylic acid
DO: Dissolved oxygen
*E. coli*: *Escherichia coli*
EDTA: Ethylenediaminetetraacetic acid
NSPs: Nonstarch polycaccharides
*P. pastoris*: *Pichia pastoris*
PMSF: Phenylmethylsulfonyl fluoride
YNB: Yeast nitrogen base
YPD: Yeast extract peptone dextrose medium
